# A simple, low cost and reusable microfluidic gradient strategy and its application in modeling cancer invasion

**DOI:** 10.1038/s41598-021-89635-0

**Published:** 2021-05-13

**Authors:** Mohamadmahdi Samandari, Laleh Rafiee, Fatemeh Alipanah, Amir Sanati-Nezhad, Shaghayegh Haghjooy Javanmard

**Affiliations:** 1 Department of Physiology, Applied Physiology Research Center, Cardiovascular Research Institute, Isfahan University of Medical Sciences, Isfahan, 81746-73461 Iran; 2 Department of Biomedical Engineering, University of Connecticut Health Center, Farmington, CT 06030 USA; 3 Center for Bioengineering Research and Education, and Department of Mechanical and Manufacturing Engineering, University of Calgary, Calgary, AB T2N 1N4 Canada

**Keywords:** Lab-on-a-chip, Biomedical engineering, Metastasis

## Abstract

Microfluidic chemical gradient generators enable precise spatiotemporal control of chemotactic signals to study cellular behavior with high resolution and reliability. However, time and cost consuming preparation steps for cell adhesion in microchannels as well as requirement of pumping facilities usually complicate the application of the microfluidic assays. Here, we introduce a simple strategy for preparation of a reusable and stand-alone microfluidic gradient generator to study cellular behavior. Polydimethylsiloxane (PDMS) is directly mounted on the commercial polystyrene-based cell culture surfaces by manipulating the PDMS curing time to optimize bonding strength. The stand-alone strategy not only offers pumpless application of this microfluidic device but also ensures minimal fluidic pressure and consequently a leakage-free system. Elimination of any surface treatment or coating significantly facilitates the preparation of the microfluidic assay and offers a detachable PDMS microchip which can be reused following to a simple cleaning and sterilization step. The chemotactic signal in our microchip is further characterized using numerical and experimental evaluations and it is demonstrated that the device can generate both linear and polynomial signals. Finally, the feasibility of the strategy in deciphering cellular behavior is demonstrated by exploring cancer cell migration and invasion in response to chemical stimuli. The introduced strategy can significantly decrease the complexity of the microfluidic chemotaxis assays and increase their throughput for various cellular and molecular studies.

## Introduction

Chemical gradients play crucial roles in vivo. Cells within different tissues sense various chemical signals and modulate their behavior accordingly^1^. Although challenging to recapitulate complex three dimensional (3D) cellular microenvironment in vitro, developing a simple but reliable biomimetic approach for generating these gradients can significantly improve our understanding about cellular behavior, cell–cell interaction and the function of their native tissue^2,3^. The traditional “Boyden chamber” is the most widely used chemotaxis device, which generates concentration gradients between two centimeter-scale wells separated by a permeable membrane^4^. Although simple, this approach (and similar traditional approaches^2^) suffer from (i) inability for monitoring cellular morphology and migration path, (ii) inaccurate control on the generation of micrometer-scale chemical signals crucial for mimicking in vivo microenvironment, (iii) consumption of a large amount of expensive bioactive factors, (iv) inability for generation of realistic 2D or 3D signals or application of more than one chemotactic factor, and (v) instability in generating long-lasting concentration gradients for long time experiments^5^.

Lab-on-chip strategies have overcome many of these challenges by manipulation of chemotactic factors in microscale channels and chambers. Microfluidic devices provide highly biocompatible microenvironment which can be used for real-time monitoring of cellular behavior^6–8^. These systems can generate small characteristic scale chemical signals with accurate spatiotemporal control to study cellular response down to single-cell level^9–11^. Small microfluidic channels further offer minimal consumption of bioactive factors which significantly reduce the experimental costs. Complex chemokine gradients can be created with microfluidic systems to investigate the effect of complex microenvironment in vivo^12,13^*.* Finally, rapid and stable generation of gradients with high resolution is easily achievable in these systems^14^.

However, application of microfluidic gradient generators for routine biomedical and biological applications faces several challenges^15^. Most of microfluidic systems require accurate active pumping for generation of chemical gradients as well as providing metabolic support for cells cultured within the device^16^. As a result, microfluidic chips are usually integrated with syringe pumps, which makes their handling complicated. To resolve this challenge, researchers attempted to incorporate reservoirs into the chip design, serving as the source of reagents, to develop pump-less microfluidic gradient generators^17,18^. While this strategy reduces the complexity of the microfluidic gradient generators, the continuous mass transfer in these systems, needed to reach equilibrium state, can affect the stability of the established gradients in the device^5^. As a result, porous materials such as hydrogels along with large reservoirs have been integrated into such devices to optimize the diffusion of chemotactic factors inside the microfluidic network, increase the stability of the gradient during the assay, and prolong the life of the generated gradient^19,20^.

Another major challenge is the need for multiple complex and time-consuming preparation steps that significantly decrease the throughput of the microfluidic assays. The preparation process is usually based on soft-lithography of PDMS-based microchannels followed by their surface treatment, bonding to glass or PDMS surfaces, multiple coating steps for cell adhesion, and in some cases, in situ formation of hydrogel barriers for generation of diffusion-based chemical gradients^21–23^. Any unexpected condition or mistake in this multi-step fabrication and preparation processes necessitates repeating the fabrication and preparation procedures from the beginning. Furthermore, multiple surface treatment and coating steps can affect the geometrical accuracy and wettability of the channels, making the device susceptible to clogging or uncontrolled solution confinement in specified channels, therefore reducing reproducibility of the assay and necessitating an extensive statistical approach^21^.

In this study, we develope a simple, low cost and reusable microfluidic gradient generator to resolve the above-mentioned challenges of microfluidic assays for gradient-based cellular studies. A stand-alone PDMS-based microfluidic chip is fabricated and directly attached to the cell culture plates without any surface pre-treatment or subsequent coating, making it detachable and reusable. The bonding strength is characterized and evaluated for injection and maintaining of solutions in the microfluidic network. Using numerical simulations and experimental examinations, the generation and stability of different chemical signals are investigated. The device is then exploited to study the behavior of cancers cells in response to biological stimuli. We believe that the developed strategy can significantly increase the simplicity, reliability and throughput of the current microfluidic gradient generator devices for cellular and molecular studies.

## Materials and methods

### Materials

SU-8 2050 and its developer were purchased from MicroChem Corp. (USA) and used for microfabrication of microfluidic device master mold on a silicon wafer obtained from Nano-BAZAR (Iran). SYLGARD^®^ 184 polydimethylsiloxane (PDMS) kit, and Tygon tubing were purchased from Dow Corning (USA). MCF7 and MDA-MD-231 breast cancer cell lines were purchased from Pasteur Institute (Tehran, Iran). Cell culture reagents including Dulbecco’s phosphate buffer saline (DPBS), Dulbecco’s modified eagle medium (DMEM), fetal bovine serum (FBS), trypsin-Ethylenediaminetetraacetic acid (EDTA), penicillin/streptomycin, and Geltrex^®^ were purchased from Gibco (Thermofisher Scientific, USA) while CellTrackers were obtained from Invitrogen (Thermofisher Scientific, USA).

### Design and fabrication of the microfluidic gradient generator

The design of the gradient generator microfluidic chip is shown in Figure S1. The design consists of (i) main culture channel, (ii) four signal channels, (iii) hydrogel channels separating signal channels from the main culture channel, and (iv) reagent reservoirs. The hydrogel channels are designed based on the capillary effect, so they offer easy and reproducible hydrogel filling while preventing the hydrogel solution from entering the signal or culture channels (Figure S1B).

The microfluidic chips were fabricated using photo-lithography and soft-lithography approaches as described previously^24^. Briefly, a master mold was fabricated by patterning SU-8 on a silicon wafer. Then PDMS base and curing agents were mixed together with a 10:1 volumetric ratio, poured on the master mold, degassed in a vacuum desiccator, and baked on a hot plate (RH digital, IKA, Germany) set at 80 °C. Different baking durations were used to optimize bonding strength of the PDMS layer to polystyrene (PS, Falcon™ tissue culture dishes and plates, Corning, USA), while 30 min was selected as the optimized value for the rest of the experiments. After baking, PDMS was cut, peeled off from the master mold and punched using 1 mm and 4 mm disposable biopsy punches (KAI instruments, Japan) to make inlets for the hydrogel and reservoirs, respectively. Before experiments, the PDMS chips were cleaned using transparent tapes, rinsed with ethanol and sterile distilled water, and stored in room temperature.

### Evaluation of PDMS/PS bonding strength

To evaluate PDMS/PS bonding strength, a PDMS-based microfluidic layer with a long square microchannel (100 μm × 100 μm) was fabricated and placed on a PS surface, followed by applying gentle pressure for removing any air between the surfaces. Then, water containing a red dye (for better visualization) was injected into the microfluidic channel at a rate of 1 μL min^−1^ using an accurate syringe pump (AL-1000, World Precision Instruments, USA). Fluid flow was monitored under an inverted microscope (Leica DM IL LED) and the length of the microchannel filled with the fluid immediately before leakage was measured. Bonding strength was calculated using the following equation^25^:

$$Bonding \;\; Strength= \left(28.4 \eta L q {h}^{-4}\right)+\left(-\gamma \left[\frac{3{\cos}{\theta }_{PDMS}+\cos{\theta }_{PS}}{h}\right]\right) (1)$$

where the first term stands for the flow resistance in the microfluidic channel and the second term indicates the capillary pressure. In this equation, $\eta$, $L$, $q$, $h$ and $\gamma$ are dynamic viscosity of water, the filled length of the microchannel, flow rate, width or height of the microchannel and surface tension of the water. Also, ${\theta }_{PDMS}$ and ${\theta }_{PS}$ are water contact angles on PDMS and PS surfaces, respectively. The contact angles were measured to be ~ 110° and ~ 80° for PDMS and PS, respectively.

### Characterization of mass transport in the microfluidic device

To characterize transport, diffusion rate and stability of a chemotactic factor, finite element simulations were performed in COMSOL Multiphysics 5.4 using “Free and Porous Media Flow” module coupled with “Transport of Diluted Species” module. A 2D model was developed corresponding to the actual microchannel dimensions and discretized with “Physics-controlled” fine triangular meshes (the mesh contained 11,974 domain elements and 1384 boundary elements). “No slip” boundary condition was considered for the fluid flow. The velocity field was first obtained by solving the model using a stationary solver (Number of degrees of freedom (DOFs) solved for were 22,161). Subsequently, the mass transport of the chemotactic factor was assessed in the pre-solved velocity field using “No flux” boundary condition and a time dependent solver (Number of DOFs solved for were 14,774, plus 4264 internal DOFs). The results were then exported and evaluated using Microsoft Excel software. A similar approach was used for the 3D simulation, with a model containing 790,008 domain elements, 96,358 boundary elements, and 7710 edge elements. The DOFs in 3D fluid flow simulations were 937,264, while the DOFs solved for the mass transport simulation were 234,316 plus 864,427 internal DOFs.

Experimentally, water containing a food red dye was injected into a signal channel and its diffusion was monitored using an inverted microscope. The bright-field microscope images were then imported into the COMSOL Multiphysics software and the intensity of the color was measured along the line passing from the center of the culture channel toward the signal channel. All data were then normalized to the intensity of the dye in the main signal channel and plotted in the combination with numerical data.

### Cell culture

Two breast cancer cell lines, including MCF7 and MDA-MD-231 were purchased from Pasteur Institute (Tehran, Iran) and cultured in DMEM, supplemented with 10% FBS and 1% penicillin–Streptomycin, at 37 °C in a humidified atmosphere of 95% air and 5% CO_2_. At 70–80% confluency, cells were washed with DPBS, harvested with 0.025% trypsin–0.01% EDTA, followed by trypsin deactivation, centrifuged at 1500 rpm for 5 min, resuspended in the new medium, and subcultured or used in the experiments.

### Preparation of the microfluidic device for cell studies

The PDMS microchips were directly mounted on the six-well cell culture plate by applying gentle pressure on the PDMS such that the channel side faced on the surface of the cell culture plate to form the sealed microchannel network. Then, 2 µl Geltrex was gently injected into each hydrogel microchannel using a 10 µl pipette. As a result of the capillary effect, Geltrex precursor was easily confined between the micro-posts designed in the hydrogel microchannels (Figure S1). By incubating the microfluidic device in a humid chamber at 37 °C for 8 min, Geltrex was transformed to the solid state and therefore the hydrogel between the micro-posts isolated the cell culture chamber from the signal channels. Culture medium (37 °C) was then pipetted into the reservoirs to fill the channels and prevent dehydration of the gel. The devices were kept inside the incubator before cell seeding.

For cell seeding, the medium from both culture channel reservoirs was removed followed by adding cell suspension (5 × 10^6^ cells/ml) to one reservoir and left to equilibrate. Due to the pressure difference, cell suspension quickly flowed toward the outlet reservoir which resulted in a uniform cell seeding. The device was then incubated at 37 °C for 2 h to allow cell attachment. The cell-containing solution in the reservoirs was then replaced with a fresh culture medium to remove excessive cells from the reservoirs. The well plate containing microfluidic devices was finally placed in a cell culture incubator and cellular behavior was monitored each day.

### Invasion assay

For evaluating the functionality of the microfluidic chips, a cell invasion assay was designed and performed within the microchips. Since the designed microchip contains four signal and one culture channels, two signal microchannels were filled with serum-free medium to serve as controls and the other two signal channels were filled with 20% FBS medium as test conditions. The cell culture channel was then filled with medium having 5% FBS. The medium in each reservoir was replaced with fresh corresponding medium every day to ensure a stable chemotactic factor gradient across the cell culture chamber during cell invasion experiments. Following the completion of cell adhesion and establishment of the chemical gradients, breast cancer cell invasion was monitored and photographed using a digital camera (Canon EOS 1300D) mounted on a phase contrast inverted microscope (Leica DM IL LED). The invasion of the cells at each selected area was quantified by measuring the change in ratio of hydrogel scaffold area occupied by cells to the total hydrogel area, using the ImageJ software. Furthermore, the directionality of the invasive cells was measured using the “Directionality” plugin, while the color maps were generated using the “OrientationJ” plugin in ImageJ.

### Statistical analysis

All tests were performed at least in triplicates and data were presented as means ± standard deviation. The comparison between the groups were performed using one- or two-way ANOVA, and data were presented as *P < 0.05, **P < 0.01, ***P < 0.001, and ****P < 0.0001, where P stands for adjusted P-value.

## Results and discussion

**Figure 1:**
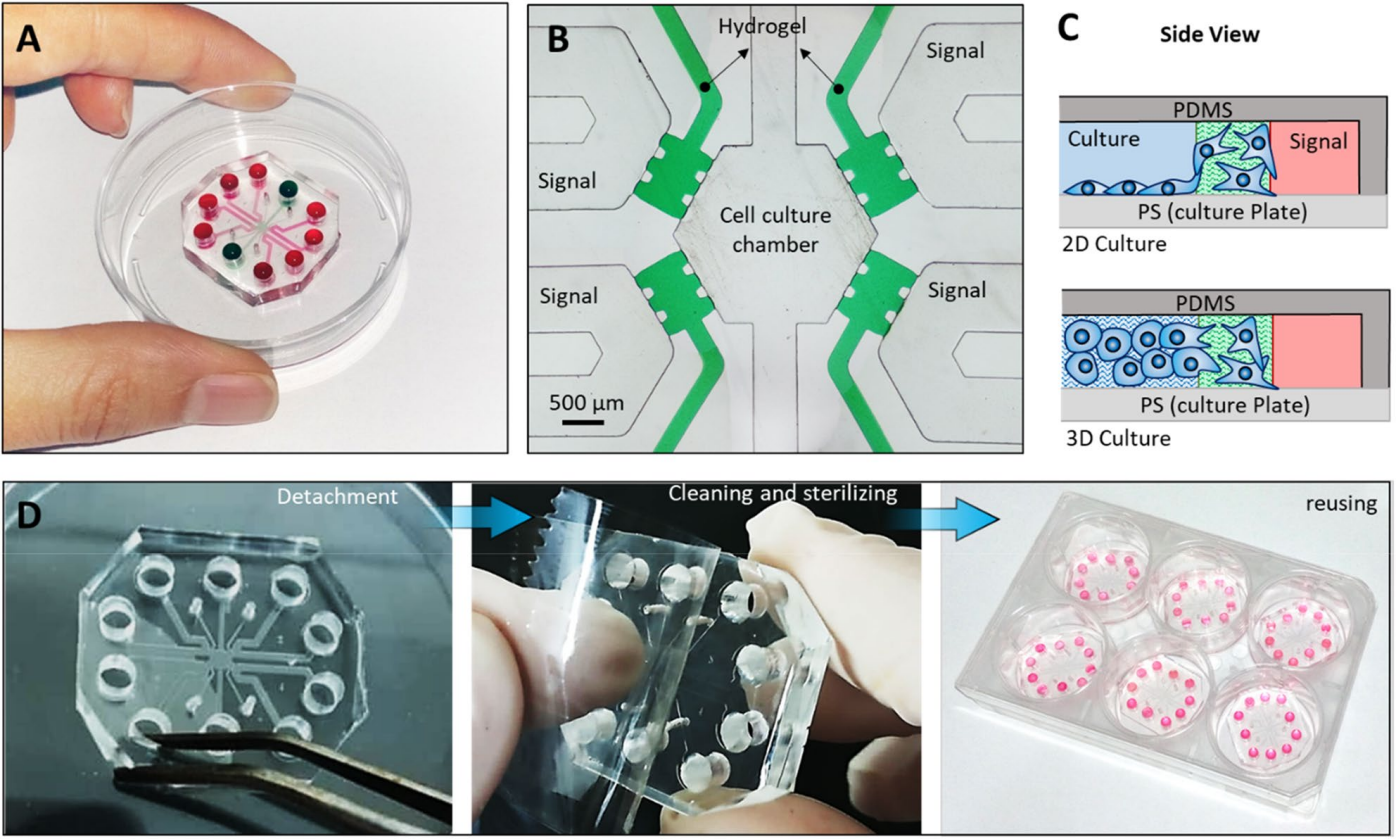
Stand-alone and reusable microfluidic gradient generator for investigation of cellular behavior. (**A**) The device is formed by direct mounting of the Polydimethylsiloxane (PDMS) microfluidic chip on the polystyrene (PS)-based culture dishes or plates. (**B**) The microfluidic network consists of four signal channels separated from a cell culture channel using hydrogel barriers. The hydrogel is confined in the specified microchannel by application of micro-posts and due to the hydrophobic nature of PDMS. (**C**) The device can be used directly for two- or three-dimensional cell culture applications and offers the opportunity for investigating cellular invasion into biomimetic extracellular matrices (ECMs). (**D**) The device is detachable and can be reused after minimal cleaning and sterilization.

To resolve the challenges associated with complicated preparation process of microfluidic gradient generators, here we introduced a simple but robust strategy by application of a stand-alone detachable microfluidic device (Fig. 1). The molded PDMS layer was directly mounted on the PS surface of the cell culture dishes (or plates) to form the sealed microfluidic cell culture system (Fig. 1A).

PDMS is the most widely used material for fabricating microchannels implemented in biomedical applications due to its easy molding, elasticity, transparency, gas permeability and biocompatibility^26,27^. After its molding, it is usually bonded to silicon-based materials, including glass and PDMS itself, to seal the microchannels and form microfluidic network^28^. However, to culture cells in these microchannels, their surfaces need to be coated or treated to support cell adhesion. Consequently, neither PDMS nor glass are preferred materials for cell culture. Polystyrene, on the other hand, is the most widely used material for culturing adherent cells due to its low cost production, transparency and easy sterilization^29^. Various surface modifications have been already optimized for PS surfaces to enable adhesion of different cell types^30^. As a result, integrating molded PDMS microchips with commercially available PS-based cell culture plates could be a significant improvement for developing ready-to-use cell culture microfluidic devices. Here, we combined a stand-alone microfluidic strategy with PDMS/PS integrated microchannels to offer a simple but robust approach for microfluidic-based cell culture systems. The stand-alone strategy enables pump-less microfluidic system by application of reservoirs, providing sufficient cell culture media, while it offers low fluid pressure in the microchannels that eliminates the requirement of strong PDMS/PS bonding.

The gradient microfluidic device used in this study consisted of four signal channels, each separated from the cell culture chamber by a hydrogel barrier (Fig. 1B). By injection of a bioactive factor in one of the signal channels, chemotactic molecules diffused through the porous hydrogel into the culture chamber and generated a chemotaxis gradient from the source signal channel to the other ones, named sink channels. Diffusion-based microfluidic gradient generators with hydrogel barriers have substantial advantages over the other systems, including (i) providing a shear-free culture system by elimination of convection, (ii) establishment of continuous chemical gradients in contrast to flow-based systems, (iii) facile fabrication without the need for multi-thickness channels, long channels or small features, and (iv) application of extracellular matrix (ECM)-based materials, offering the opportunity for performing biomimetic functional assays such as cellular invasion assay.

The proposed system in this study overcomes the problems frequently happening in preparation of similar microchips^21^. First, due to the implementation of PS as the substrate of the microchannels, our system does not require subsequent coating and therefore it is not limited to specific cell types. Cells can be either directly seeded inside the microchannels, forming a 2D culture system, or encapsulated in a hydrogel for their more realistic 3D culture (Fig. 1C). Second, uniform and reproducible surface condition ensures uniform cell seeding and reliable results. Third, the hydrophobicity of the microchannel walls is not disrupted by plasma treatment or coating, and therefore hydrogel confinement in specified microchannels is easy. Finally, the system is detachable, which makes the device reusable and omits the time- and cost-consuming fabrication steps (Fig. 1D). Using a brief tape cleaning followed by sterilization, the microchips can be mounted again in well plates and used in the experiments.

### Optimizing PDMS/PS bonding strength

**Figure 2:**
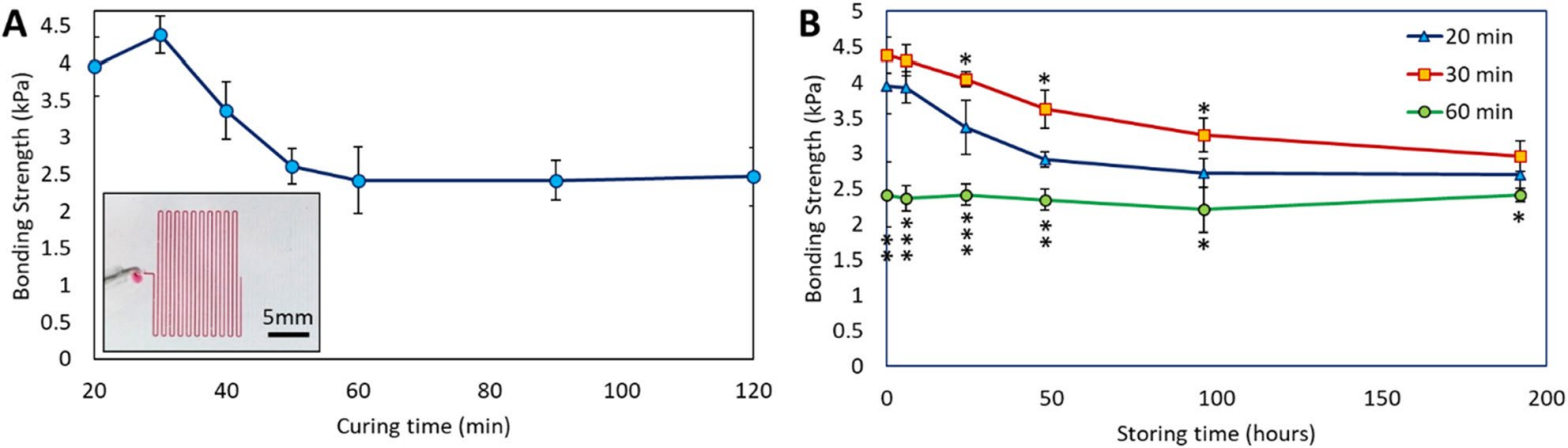
Characterization of PDMS/PS bonding. (**A**) Optimization of bonding strength by changing the PDMS curing time. Inset shows the microfluidic strategy used to evaluate the bonding strength. (**B**) PDMS/PS bonding strength by aging the PDMS microchip, having specific initial curing durations (20, 30 and 60 min). The results of statistical analysis of the bonding strength with 30 min baking compared to 20 min baking are shown above the 30 min graph, while the results comparing the 30 min and 60 min are shown below the 60 min graph (*P < 0.05, **P < 0.005) *P < 0.05, **P < 0.005, ***P < 0.0005).

The assessment of PDMS/PS bonding was performed to ensure fabrication of a leakage-free microfluidic device (Fig. 2). It is well-known that surfaces with minimal roughness can adhere to each other as a result of attractive forces, such as short-range van Der Waals force, to decrease their surface energy^31^. In the case that at least one of the surfaces is deformable, it can conform to the macro- and micro-features usually present on the target surface and increase the intimate contact area, which consequently enhances the adhesion force^32^. The elasticity of PDMS was exploited here to form a stable bond to PS and create a leakage-free microfluidic device. Additionally, surface charge of the PDMS layer generated after tape-cleaning could improve the bonding strength^33^. The bonding strength of PDMS to PS was measured using a microfluidic strategy (Fig. 2A inset). Dye-containing water was injected into a microfluidic device with predefined channel dimensions and controlled flow rate. The bonding strength was evaluated based on the flow resistance in the device. The results show that increasing PDMS curing time generally decrease the bonding strength. This result is in the agreement with previous finding which reported enhanced adhesion by decreasing the stiffness of PDMS-based structures^34^. Decreased curing time reduces the stiffness of the PDMS structure and enhances compliant contact area and therefore adhesion strength. However, insufficient curing can cause improper crosslinking. An optimum curing time is required for strong and reliable PDMS/PS bonding. Such bonding can sustain more than 4 kPa fluidic pressure, which is much more than the required amount for stand-alone microfluidic devices (a reservoir height of > 40 cm can be used).

We further measured the PDMS/PS bonding strength over time (Fig. 2B). Although the bonding strength decreased by aging the PDMS microchip, it remained in a reasonable range to be applied for stand-alone microfluidic devices. Our observations demonstrated that the device could be reused as long as it could be cleaned and there was no scratch created on the channels. The presence of scratches disturbs the designated fluid network inside the channels. The scratches can also significantly reduce the bonding strength by decreasing the intimate contact area and generating stress concentration spots, making the bond susceptible to debonding caused by small disturbances in the system. Our devices have been reused more than 30 times over a period of more than 6 months without any leakage.

Another concern for reusability of the devices is the PDMS contamination with absorbed molecules from the culture media. It has been demonstrated that PDMS can absorb a significant amount of small hydrophobic molecules^26^. However, the reversible nature of the absorption^35^ provides the opportunity for reusing the devices after proper cleaning. Given the small thickness of the PDMS devices (3–4 mm), a set of ethanol and distilled water rinsing (15–20 min each), followed by incubation in room temperature for 24–48 h can remove the majority of the contamination. The device can also be autoclaved for further removing/degrading the contaminating molecules. Our experiments showed that the device can work leakage-free after autoclaving.

### Characterization of chemical gradient signal

**Figure 3:**
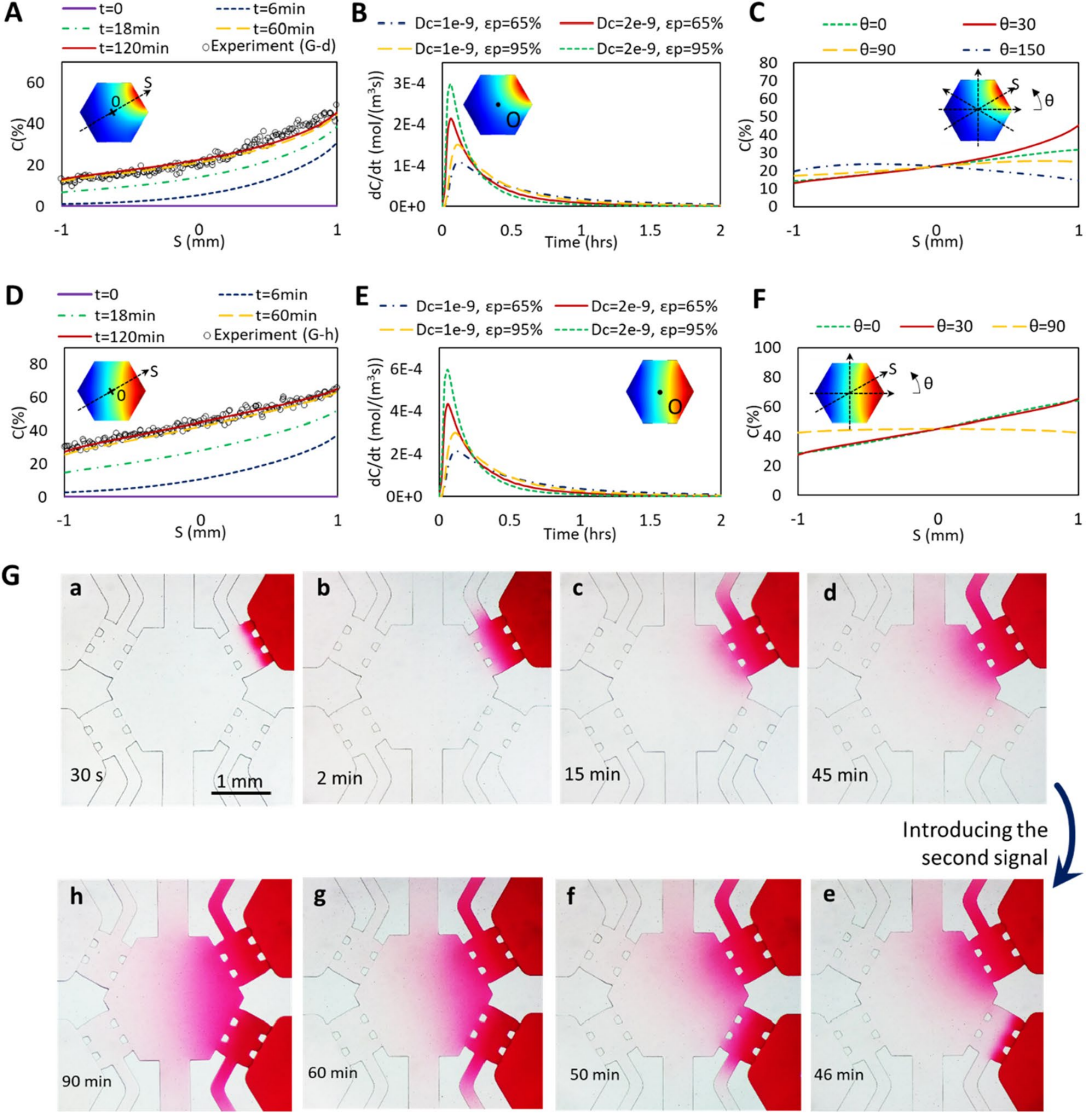
Characterization of chemotactic signal dynamics and profile. (**A**–**F**) Finite element simulations demonstrating the kinetics and shape of gradient signal along the S axis (from sink to source channels). Both polynominal (**A**) and linear (**D**) signals could be generated by introducing one or two chemotactic factors, respectively. (**B**,**E**) Concentration kinetics within the culture chamber for polynominal (**B**) and linear (**E**) signals. Concentration kinetics was measured at point O with different diffusion coefficient (Dc) and porosity (εp) of the hydrogel. In all of the conditions, the system was stabilized after ~ 2 h. (**C**,**F**) Concentration gradient profile in different directions for polynominal (**C**) and linear (**F**) signals. The largest gradient was observed at θ = 30°. (**G**) Experimental representation of chemical signal evolution (a to h) for forming different chemical gradients. To compare with the simulation results, the intensity of the dye was measured along the line passing from the center of the culture channel toward the signal channel after 45 min (**G**-d) and 90 min (**G**-h), plotted in the corresponding graphs in (**A**) and (**D**), and labeled with Experiment (**G**-d) and Experiment (**G**-h), respectively.

We further assessed the kinetics and shape of chemotactic signal in the microfluidic device using numerical and experimental evaluations (Fig. 3). The results showed that chemotactic species can diffuse from the signal channel through the hydrogel porous structure and generate a stable chemical gradient in the cell culture chamber after ~ 2 h (Fig. 3A). While the diffusion rate of the chemotactic factor depends on the porosity of the hydrogel and its diffusion coefficient, the simulation results indicated that a relatively stable concentration gradient could be generated after 2 h within a wide range of hydrogel porosities and diffusion coefficients (Fig. 3B). In is notable that the signal was measured along a line passing from the center of the culture channel toward the signal channel (Fig. 3A, inset). Figure 3C demonstrates that the strongest gradient signal is established along this line. Preserving a negligible volume of fluid stored in the cell culture chamber compared to the reservoirs volume (< 0.3%) ensures a gradient of the signal with high stability. Although the stability of the generated signals could still be a challenge of this microfluidic device in long-term experiments (more than 24 h), the time window provided by reservoirs combined with daily replacement of reservoir solutions with fresh media can ensure the stability of the signals during the whole assay. A full-model 3D simulation was performed over a 24-h period to further confirm the presence and stability of the concentration gradient generated within the cell culture chamber between the daily refreshment of the solutions. While a dynamic mass transport is always available in every static gradient generator until the formation of an equilibrium, the unique design of the microfluidic system in this work (with embedded hydrogel barriers and large reservoirs) caused the formation of a stable gradient with a negligible decrease in the gradient magnitude after the first 2–4 h of the gradient generation (Figure S2).

We further examined the capability of the device to produce different chemical gradient profiles (Fig. 3D–F). It has been demonstrated that cellular behavior changes with the shape of gradient profile^36^. To address this requirement, we showed that the developed device is able to generate both polynomial (Fig. 3A) and linear (Fig. 3D) signals with high accuracy (> 99.8%). The quantitative (Fig. 3A,D) and qualitative (Fig. 3G) representation of experimental evaluations of chemotactic signal dynamics in the system further demonstrated good agreement with numerical results.

### Case study: evaluating cancer cell behavior

**Figure 4:**
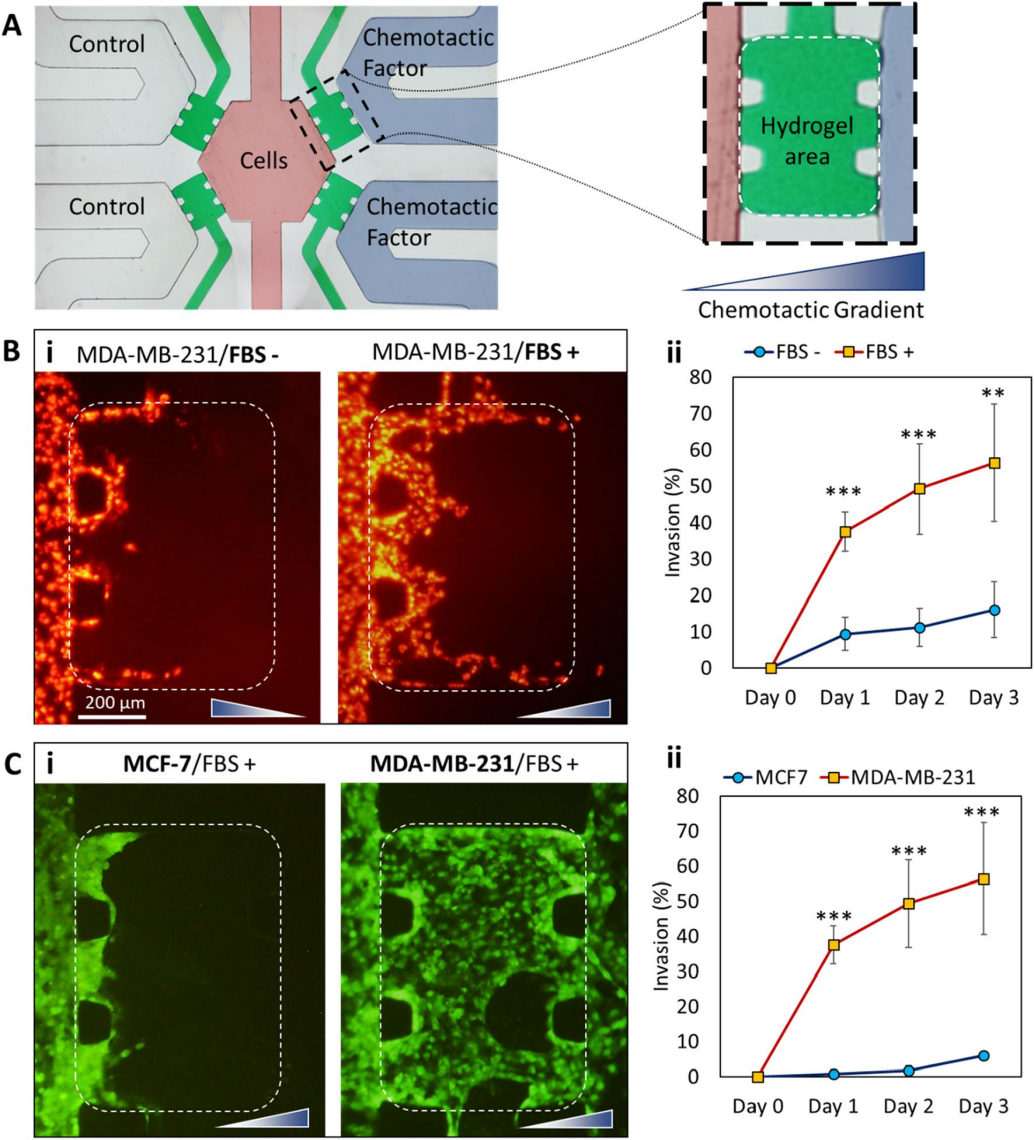
Investigating the invasion of different cancer cells in response to different chemotactic factors. (**A**) The border of hydrogel regions is indicated by dashed white lines. (**B**) MDA-MB-231 breast cancer cell invasion in response to additional FBS in signal channels, two days post-seeding. (**C**) Comparison of two breast cancer cell line invasiveness, using the proposed microfluidic strategy, three days post-seeding. In both (**B**) and (**C**) subfigures, representative images are shown in (i) while the quantitative evaluations are shown in (ii). The direction of the gradient is shown by triangular gradient shape at the bottom-right of each image. ***P < 0.0005.

To demonstrate the potential of the developed microfluidic strategy for capturing cellular behavior, the invasion of breast cancer cells into an ECM-based material was investigated under specific chemical gradients (Fig. 4). Geltrex was used as the model ECM and FBS was considered as the chemoattractant. Two signal channels were filled with growth medium supplemented with 20% FBS (v/v) while FBS-free medium was introduced to the other signal channels to serve as controls (Fig. 4A). Application of four signal microchannels enabled the testing conditions and control experiments to be performed simultaneously in the same device. Therefore, cell migration/invasion toward the channels containing chemotactic factor or the control channels could be directly compared (Fig. 4B). The result showed that the microfluidic strategy supports cellular attachment, proliferation and migration/invasion, without the requirement of any coating procedure.

As expected, the invasion of cancer cells into the ECM was enhanced by application of higher FBS concentration in signal channels (Fig. 4B). The application of a hydrogel between the cell culture chamber and signal microchannels mimics the native in vivo cellular microenvironment and offers the feasibility of a functional assay for investigating cancer cell invasion^37,38^. The results confirmed that the functionality of different cell lines was well preserved. Using two different breast cancer cell lines including very invasive MDA-MB-231 compared to less-invasive MCF7 cells^39^, we observed significantly less invasion (56% ± 16% vs 6% ± 1%) in the experiments with MCF7 cells (Fig. 4C). This is in accordance with previous investigations^40^, suggesting that MCF7 cells are unable to affect the integrity of ECM, while the highly invasive MDA-MD-231 cells can easily degrade and infiltrate into it.

Cancer metastasis is governed by tumor cells intravasation into the circulation, followed by their extravasation from the circulation, to form a secondary tumor^37^. Both intravasation and extravasation involve invasion of cancer cells into ECMs. The invasion of cancer cells is accomplished through the degradation of ECM, induced by gradients of chemotactic factors^40^. The presence of matrix metalloproteinase (MMP) degradation sequences in the ECM-based scaffolds allows the cells to degrade the scaffold through proteolytic action^41,42^. When cancer cells can establish a strong cellular communication, the invasion of the leader cells into the ECM can be followed by migration of other cancer cells through the conduits formed by the leader cells. This migration is modulated by the chemical gradient present in the microenvironment, causing a “collective cancer cell migration”^43^. A similar behavior was observed in the invasion assays of MDA-MB-231 cells in this work. Leader cells degraded the ECM and formed narrow conduits through the ECM hydrogels, followed by migration of other cells through the generated conduits (Figure S3). As a result of such cellular behavior, the invasion rate decreased over time while a complete (100%) invasion was never observed in the experiments (Fig. 4B ii and C ii). After the initial dates of the invasion assay, leader cells completely pass the ECM barrier and enter the signal channel, leaving behind the formed conduits. Subsequent migration of the other cells through the ECM conduits lowers the requirement of further cell invasion, because the cells favor facile migration over ECM degradation to reach the chemoattractant source. As a result, some regions of ECM were left intact (Figure S3). Similar to in vivo microenvironment^44^, both primary invasion and secondary migration are induced by the presence of chemoattractant gradient in the microfluidic device. The collective cancer cell migration further confirms the presence of a strong signal communication between MDA-MB-231 cells in microfluidic device. It has been demonstrated that collective cell migration has a higher invasive capacity and higher resistance to clinical treatments than the single tumor cell migration^43^. Furthermore, our results indicated a high invasion potency at the interfaces of the hydrogel with microchannel walls, even in MCF7 cancer cells (Figure S4). In most of cell studies, invasion started from the interfaces of the ECM and channel walls. This is in accordance with previous investigations^45^ reporting that the presence of interface plays an important role in guiding cellular invasion.

**Figure 5:**
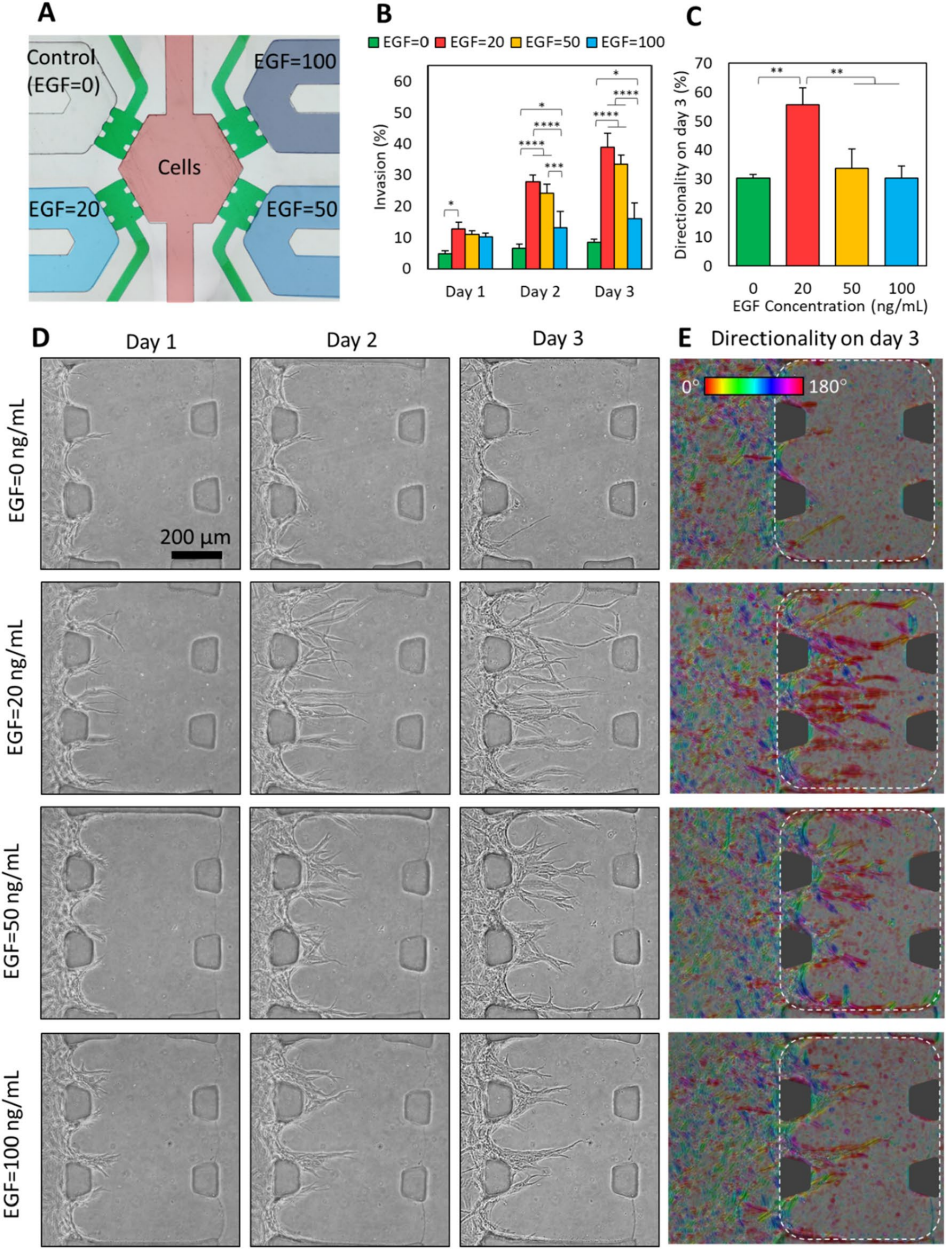
The dose-dependent response of the MBA-MD-231 cells to EGF gradients. (**A**) The design of the experiment in each microfluidic device. The numbers represent the concentration of the EGF in ng/mL. (**B**) The invasion levels of MBA-MD-231 cells in response to different EGF gradients. The invasion levels were measured from the area of ECM occupied by the cells. (**C**) The directionality of the invasive cells at day 3 post-seeding, in response to different EGF gradients, obtained using ImageJ software. (**D**) Representative images of each gradient section of a single device on different days post-seeding. (**E**) Qualitative representation of the cellular directionality. A color map is generated using ImageJ software to demonstrate the random orientation of the cells in the cell culture chamber and their directionality in the ECM section. A reddish color indicates the direction of the invasion aligned with the concentration gradient direction. For a better comparison, all figures rotated to position the cell culture chamber at the left and EGF signal channel at the right side of the image. The whole device that is used in (**D**) and (**E**) is shown in Figure S5.

To further validate the potential of the device for chemotaxis assays, we investigated the effect of epidermal growth factor (EGF) gradients on MDA-MB-231 cell invasion (Fig. 5). As shown in Fig. 5A, different concentrations of EGF (0 to 100 ng/mL) were introduced in different signal channels of the microfluidic device. Interestingly, a dose-dependent behavior was observed in the invasion of the MDA-MB-231 cells, with a maximum invasion detected at 20 ng/mL EGF concentration gradient (Fig. 5B,D). This dose-dependent behavior of MDA-MB-231 cells is in accordance with previous reports^46,47^. We detected a dose-dependent change in the directionality of the cells and consequently in the invasion type (Fig. 5C,E). The highest directional invasion and therefore collective cell migration potency was detected for the cells subject to 20 ng/mL EGF concentration gradient. While a significant difference could not be detected in the level of invasion (area of the ECM occupied by the cells) between the cells affected by 20 ng/mL gradient compared to those affected by 50 ng/mL gradient (Fig. 5B), the directionality in the cells experiencing 20 ng/mL gradient signal was significantly higher than all the other groups (Fig. 5C). This shows the capability of performing multiplex bioassays using a simple and reusable microfluidic strategy, emphasizing the benefit of the strategy for research and clinical applications.

## Conclusion

To overcome complexities associated with preparation and application of microfluidic gradient generators for investigating cellular behavior, here we developed a rapid, simple and cost-effective strategy. A stand-alone microfluidic strategy combined with PDMS/PS integrated microchannels was employed to offer a pumpless, detachable and reusable microfluidic cell culture assay. Direct mounting of the molded PDMS layer onto the surfaces of PS plates eliminated the requirement of any surface treatment or coating for cell adhesion. The bonding strength of the PDMS to PS was optimized and demonstrated to be strong enough for establishment of a leakage-free stand-alone microfluidic cell culture system. By decreasing the stiffness of the molded PDMS layer, bonding strength was increased due to better compliance of the contact surfaces. A diffusion-based chemical gradient generator was formed using hydrogel barriers separating cell culture chamber from the signal microchannels. Numerical simulations and experimental evaluations further were performed to characterize the transport of chemotactic factors through the hydrogel network via diffusion mechanism. The results indicated that the proposed design of microfluidic network offers reliable formation of both linear and polynomial chemical signals. To assess the feasibility of the developed device for investigating cellular behavior, the device was implemented to investigate cancer cell migration and invasion. The device reliably supported cellular growth, proliferation and migration of breast cancer cells. The incorporation of hydrogel scaffold further enabled monitoring cellular invasion. We demonstrated that the microfluidic strategy enables differential invasion of cancer cells in response to generated chemical signals, while supporting the cellular behavior and functionality. This simple and rapid strategy can decrease the complexities associated with preparation and application of microfluidic-based cell culture systems.

While this study describes the development and optimization of a robust but simple microfluidic strategy for multiplex evaluation of cellular behavior in response to various chemical gradients, the current work can be continued in future studies: (i) different chemotactic factors can be introduced in a single device to assess the interaction of various factors and their effects on the cellular behavior; (ii) cell-laden hydrogels can be used in different channels of the device to reproduce a more realistic 3D cellular environment similar to in vivo conditions; and (iii) the microfluidic network used here can be multiplicated into an integrated chip to enhance the throughput of the strategy for large chemotaxis studies with multiple variables.

## Supplementary Information

Supplementary Information.
