# Supplementary material for: A simple, low cost and reusable microfluidic gradient strategy and its application in modeling cancer invasion
Source: Sci Rep. 2021 May 13;11:10310. doi: 10.1038/s41598-021-89635-0 (PMC8119451; doi:10.1038/s41598-021-89635-0)
Supplement: Supplementary file 1 — Supplementary Information. [file 41598_2021_89635_MOESM1_ESM.pdf]

Supplementary information

A simple, low cost and reusable microfluidic gradient strategy and its application in modeling cancer invasion

Mohamadmahdi Samandari[a,b,1], Laleh Rafiee[a,1], Fatemeh Alipanah[a], Amir Sanati-Nezhad[b,*], Shaghayegh Haghjooy Javanmard[a,*]

[a] Department of Physiology, Applied Physiology Research Center, Cardiovascular Research Institute, Isfahan University of Medical Sciences, Isfahan 81746-73461, Iran

[b] Center for Bioengineering Research and Education, and Department of Mechanical and Manufacturing Engineering, University of Calgary, Calgary, AB T2N 1N4, Canada

* Corresponding authors: sh_haghjoo@med.mui.ac.ir (S.H.-J.); amir.sanatinezhad@ucalgary.ca (A.S.-N.); Tel.: +98-31-3669-2836 (S.H.J.); +1-(403)-220-7708 (A.S.-N.)

[1] These authors contributed equally to this work

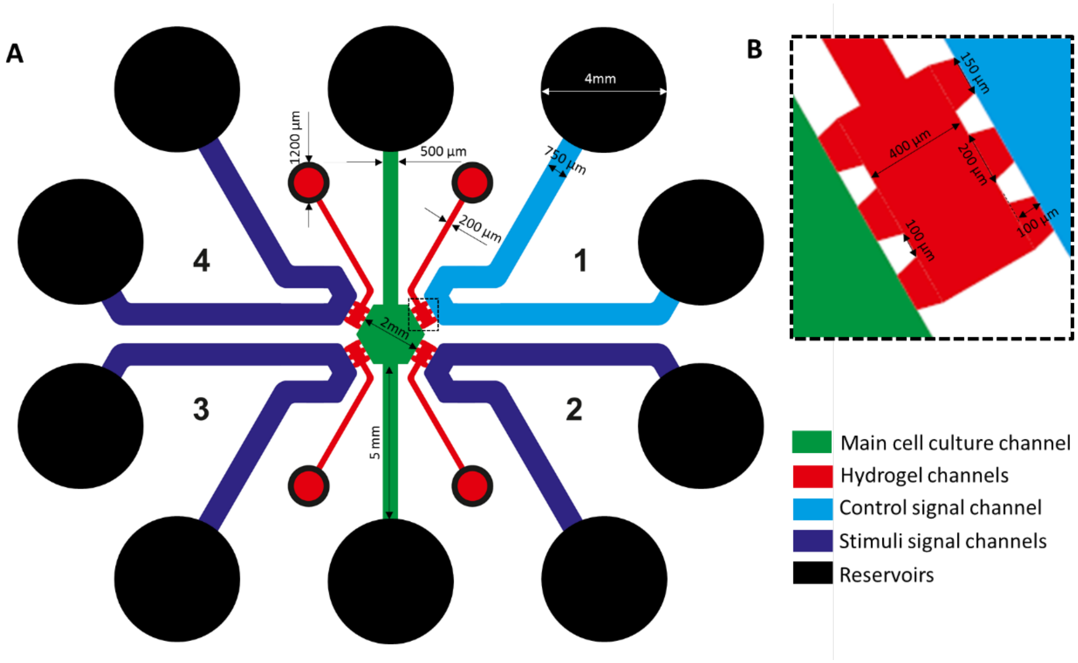

Figure S1. Schematic representation of the design of microfluidic network used in this study.

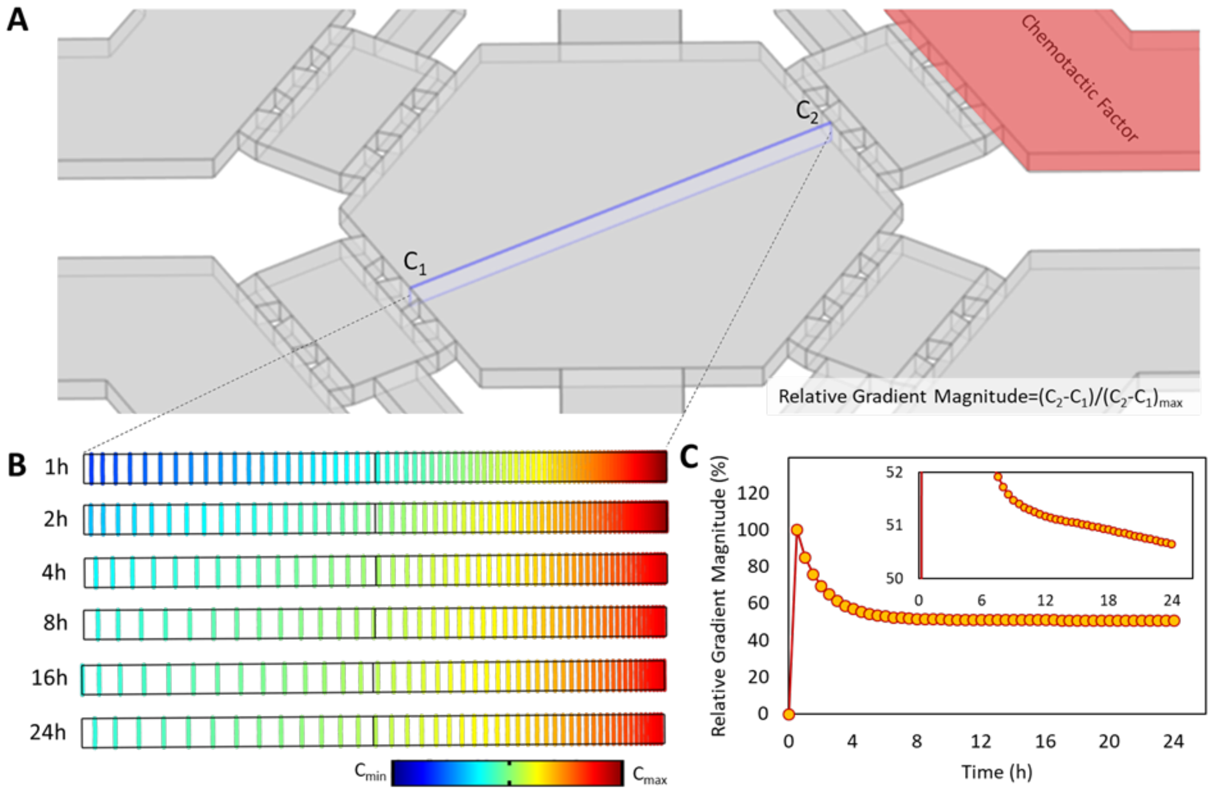

Figure S2. Full-model three-dimensional (3D) computational fluid dynamics simulation of the microfluidic system for evaluating the gradient stability between the daily fluid refreshments. (A) A chemotactic factor is introduced in one of the signal channels and the gradient is evaluated across the chamber on the plane

shown by blue color. (B) The contour map of the concentration across the cell culture chamber in different time points. While the concentration dynamically changes during the first 2 hrs, an almost stable gradient is observed in time range of 4-24 hr. (C) Quantitative evaluation confirmed the stability of the gradient over 24-hour period. As expected, a dynamic mass transport is still detectable in the system (inset), although It is a negligible amount (<2%).

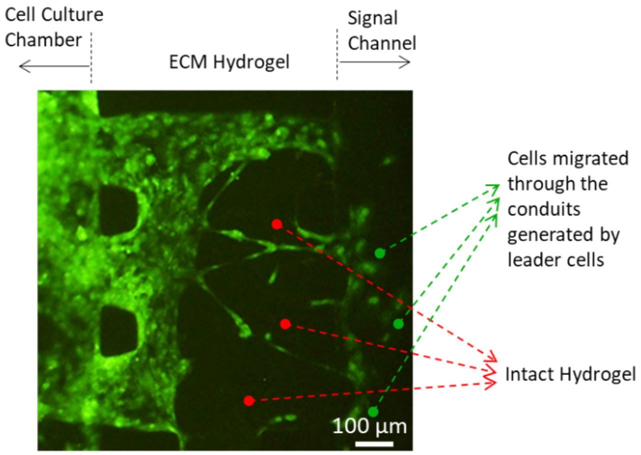

Figure S3. Collective cancer cell invasion in microfluidic gradient generator developed in this study. The invasion of MDA-MB-231 cancer leader cells by degrading the hydrogel ECM is followed by the migration of other cells through the channels generated by leaded cells.

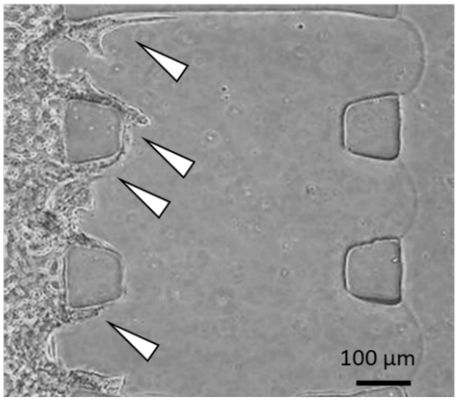

Figure S4. The effect of morphological interfaces on cancer cell invasion. The presence of interfaces between the ECM and PDMS channel walls facilitates the invasion of MCF7 cancer cells with low invasion potency. Arrow heads show the invading cells through the interfaces.

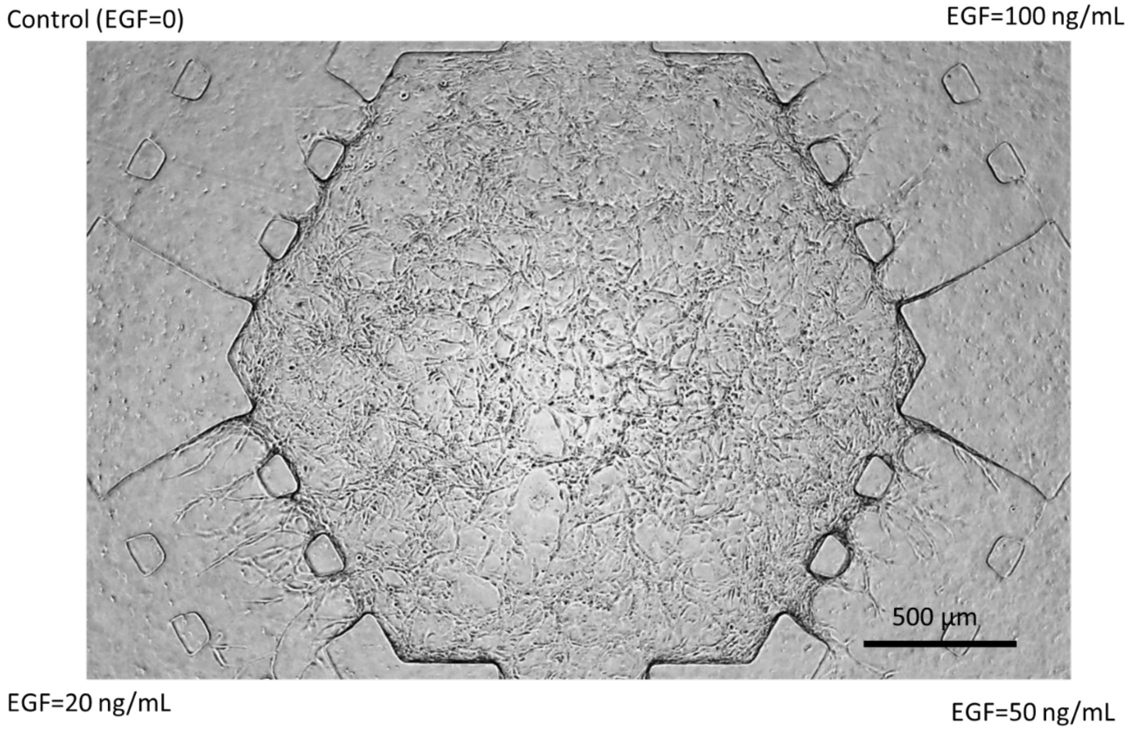

Figure S5. Demonstrating the dose-dependent response of MBA-MD-231 cells to EGF gradients in a single microfluidic device. The image is captured on day 3 post-seeding.
